# Invasive *Candida* Infection in Patients With Bacterial Infective Endocarditis

**DOI:** 10.1093/ofid/ofae179

**Published:** 2024-03-26

**Authors:** Travis Combs, Bobbi Jo Stoner, Parker McCoy, Hassan Reda, Michael Sekela, Sami El-Dalati

**Affiliations:** Department of Internal Medicine, University of Kentucky College of Medicine, Lexington, Kentucky, USA; Department of Pharmacy, University of Kentucky College of Pharmacy, Lexington, Kentucky, USA; Department of Pharmacy, University of Kentucky College of Pharmacy, Lexington, Kentucky, USA; Division of Cardiothoracic Surgery, Department of Surgery, University of Kentucky College of Medicine, Lexington, Kentucky, USA; Division of Cardiothoracic Surgery, Department of Surgery, University of Kentucky College of Medicine, Lexington, Kentucky, USA; Division of Infectious Diseases, Department of Internal Medicine, University of Kentucky College of Medicine, Lexington, Kentucky, USA

**Keywords:** antimicrobial therapy, bacterial endocarditis, *Candida* infection, injection drug use

## Abstract

Over 21 months, 12 patients with invasive *Candida* infections detected during the course of treatment of bacterial endocarditis, including 11 with candidemia, were identified. Invasive *Candida* infections can occur as a complication of bacterial endocarditis and may occur more frequently in patients with injection drug use and broad-spectrum antibiotic exposure.

Infective endocarditis (IE) is a complex disease entity that carries significant morbidity and mortality. The vast majority of cases are caused by bacterial microorganisms. Approximately 1%–6% of IE is caused by fungal pathogens, and *Candida* species account for half of these cases with an associated mortality of >30% [1, 2]. Injection drug use, presence of a prosthetic valve or cardiac device, and chronic indwelling venous access are known risk factors for fungal IE [1]. While prior endocarditis has been associated with increasing the long-term risk for subsequent episodes of *Candida* IE, the median duration between infections was 426 days in that study [3]. In 1964 Henderson and Nickerson reported the only 2 cases of *Candida albicans* superinfection in patients with bacterial IE, although in 1 patient the *Candida* and bacterial organism were isolated simultaneously [4]. Since then there has been little literature on the topic of *Candida* infections occurring in patients undergoing treatment for bacterial IE. Given the potential devastating effects of missed or delayed diagnosis of candidemia, it is crucial for providers to be aware of this phenomenon and to identify at-risk patients. To further understanding of this disease process, we report our experience with the largest case series of invasive *Candida* infections diagnosed in patients with bacterial IE.

## METHODS

Institutional review board exemption was obtained from the University of Kentucky. Cases of candidemia in the first 12 weeks after diagnosis of bacterial IE were identified using the institution's multidisciplinary endocarditis team (MDET) database. All patients discussed by MDET who developed positive blood or tissue cultures with a *Candida* species after originally being diagnosed with bacterial endocarditis were included. Detailed retrospective chart review was then performed by all of the study investigators. Twenty-seven demographic and clinical variables were recorded, including patients’ Pitt bacteremia score, Charlson Comorbidity Index (CCI) score, modified Duke criteria, and Duke–International Society of Cardiovascular Infectious Diseases (ISCVD) criteria (Table 1) [5–7]. For continuous variables, descriptive statistics were utilized.

**Table 1.** Twelve Cases of Invasive *Candida* Infections Diagnosed in Patients Undergoing Treatment for Bacterial Infective Endocarditis

Characteristic	Case											
	1	2	3	4	5	6	7	8	9	10	11	12
Age, y	31	30	59	23	42	39	31	45	41	38	40	47
Gender	F	M	F	F	M	M	F	M	M	M	M	F
Previous *Candida* infection	No	No	No	No	No	No	No	No	No	No	No	No
Previous IE (treatment)	Yes (bacterial, medical)	Yes (bacterial, medical)	No	No	Yes (bacterial, medical)	No	Yes (bacterial, medical)	Yes (bacterial, medical)	Yes, (bacterial, medical)	Yes (bacterial, medical)	No	No
Initial organism	MSSA	MSSA, *Streptococcus anginosus*	MSSA, *Klebsiella pneumoniae*	MRSA, *Streptococcus pyogenes*, *Enterobacter cloacae*, *Serratia marcescens*	MRSA	MRSA	MSSA	MRSA	MSSA	MSSA	MSSA	*Enterococcus faecalis*
Pitt bacteremia score	4	2	5	2	0	0	0	2	2	0	0	3
CCI score	0	0	6	0	0	0	0	0	0	0	1	3
Other comorbidities	Hepatitis C, anxiety, depression	NA	Cirrhosis, CKD, seizure disorder	Hepatitis C	Hepatitis C, hypertension	Hepatitis C, hypertension	Hepatitis C	Hepatitis C, intracranial hemorrhage	NA	Hepatitis C	Insulin-dependent DM	NA
Duke criteria	Definite IE	Definite IE	Definite IE	Definite IE	Definite IE	Definite IE	Definite IE	Definite IE	Definite IE	Definite IE	Definite IE	Definite IE
Duke-ISCVD criteria	Definite IE	Definite IE	Definite IE	Definite IE	Definite IE	Definite IE	Definite IE	Definite IE	Definite IE	Definite IE	Definite IE	Definite IE
Complicating factors	Pleural effusion, *Acinetobacter* pneumonia	Pyelonephritis, COVID-19	COVID-19, Influenza B	Pregnancy, pulmonary embolism	NA	Vertebral osteomyelitis	Discitis, AKI	COVID-19, intracranial hemorrhage	AKI	Sterno-clavicular septic arthritis, pulmonary emboli	Intracranial hemorrhage, pulmonary emboli, empyema, *Pseudomonas* bacteremia, iliopsoas abscess	NA
Duration of bacteremia	5 d	10 d	1 d	17 d	4 d	7 d	4 d	7 d	6 d	5 d	5 d	2 d
History of IDU	Yes	Yes	No	Yes	Yes	Yes	Yes	Yes	Yes	Yes	Yes	Yes
*Candida* species	*tropicalis*	*glabrata*	*glabrata*	*albicans, parapsilosis*	*parapsilosis*	*glabrata*	*albicans*	*parapsilosis*	*albicans*	*glabrata*	*albicans*	*parapsilosis*
Timing of *Candida* infection	Day 1	Day 12	Day 8	Day 70	Day 1	Day 3	Day 7	Day 30	Day 4	Day 2	Day 31	Day 3
Candidemia complications	No	No	No	No	No	Pan-ophthalmitis of right eye	No	Intracranial hemorrhage	No	No	No	No
Native/prosthetic valve	Native	Native	Native	Native	Native	Native	Native	Native	Native	Native	Native	Native
Affected valve (vegetation size in cm if available)	Mitral	Tricuspid (2)	Tricuspid	Tricuspid	Tricuspid	Tricuspid	Tricuspid (2.7 × 1.9)	Tricuspid and mitral	Tricuspid and aortic (1.2 × 0.9)	Tricuspid (3 × 0.9)	Tricuspid (1.5 × 0.6) and mitral	Mitral and aortic
Antibiotic treatment prior to candidemia	Nafcillin, then cefazolin	Cefazolin	Pip-Taz and tazobactam, then cefazolin	Vancomycin and cefepime	Vancomycin	Vancomycin and ceftaroline	Vancomycin and cefepime, then cefazolin and ertapenem	Vancomycin and ceftaroline	Nafcillin, then cefazolin and ertapenem	Ceftriaxone and vancomycin, then nafcillin	Vancomycin and Pip-Taz, then cefazolin and ertapenem, then cefepime	Ceftriaxone and ampicillin
Duration of therapy prior to *Candida* infection	2 d	10 d	4 d	42 d	2 d	2 d	7 d	31 d	7 d	1 d	30 d	3 d
Antifungal therapy (dose/day)	Micafungin 150 mg, then fluconazole 400 mg PO	Micafungin 150 mg	Micafungin 150 mg	Liposomal amphotericin 310 mg, then micafungin 150 mg, then PO fluconazole 400 mg	Micafungin 150 mg, then fluconazole 400 mg PO	Micafungin, then voriconazole 400 mg	Micafungin 100 mg, then fluconazole 400 mg PO	Liposomal amphotericin 390 mg, then fluconazole 400 mg PO	Micafungin 150 mg, then isavuconazole 372 mg PO	Micafungin 100 mg	Micafungin 150 mg, then fluconazole 400 mg PO	Micafungin 150 mg, then amphotericin 420 mg and flucytosine 8000 mg, then fluconazole 600 mg
Duration of candidemia	1 d	3 d	1 d	8 d	2 d	2 d	1 d	NA	6 d	1 d	3 d	8 d
Duration of antifungal therapy	18 d IV, then PO for 3 mo	14 d	6 d	56 d IV then PO indefinitely	42 d IV then PO indefinitely	19 d	42 d planned (left after 1 wk on PO)	14 d IV, then PO	30 d IV, then PO indefinitely	38 d	17 d IV, then PO indefinitely	12 d IV, then PO indefinitely
Valve surgery	Yes	No	No	No (underwent open pulmonary embolectomy)	No	No	No	No	Yes	No	No	No
Tissue culture/pathology	Valve culture and pathology negative for fungus	NA	NA	Embolism culture positive for *C albicans*	NA	NA	NA	Cranial bone culture positive for *C parapsilosis*	Valve culture positive for *C albicans,* pathology negative	NA	NA	NA
In-hospital mortality	Alive	Alive	Died	Alive	Alive	Alive	Alive	Alive	Alive	Alive	Alive	Alive
90-d mortality	Alive	Alive	NA	Died	Alive	Alive	Alive	Alive	Alive	Alive	Alive	Alive

Abbreviations: AKI, acute kidney injury; CCI, Charlson Comorbidity Index; COVID-19, coronavirus disease 2019; DM, diabetes mellitus; F, female; IDU, injection drug use; IE, infective endocarditis; ISCVD, International Society of Cardiovascular Infectious Diseases; IV, intravenous; M, male; MRSA, methicillin-resistant *Staphylococcus aureus*; MSSA, methicillin-susceptible *Staphylococcus aureus*; NA, not applicable; Pip-Taz, piperacillin-tazobactam; PO, oral.

### Definitions

Previous *Candida* infection was defined as any documented *Candida* infection within the electronic medical record of the University of Kentucky. Superficial infections, such as cutaneous *Candida* infections or vulvovaginal candidiasis, were excluded. Initial organisms were defined as the pathogens found in the first positive blood culture during the index admission. Duration of bacteremia was determined from the first positive to the first documented negative blood culture, including cultures from outside hospitals. History of injection drug use (IDU) was defined as any documented history of IDU within 30 days of admission. Timing of candidemia was determined by the number of days between the first positive bacterial blood culture and the first positive blood culture for *Candida*. Candidemia complications were defined as foci of infections that occurred after the onset of candidemia. Antibiotic treatment prior to candidemia was defined as any antibiotic given for the diagnosis of IE and prior to the onset of candidemia. Duration of therapy prior to candidemia was defined as the days of antibiotic therapy for IE prior to the first culture positive for *Candida*. Duration of candidemia was defined as the time between the first positive culture for *Candida* and the first documented negative blood culture.

## RESULTS

Between 7 September 2021 and 9 June 2023, 12 patients with bacterial endocarditis and *Candida* infections were identified from a total of 234 patients with definite endocarditis. The median age was 39.5 years (range, 23–59 years) and 7 patients were male (Table 1). Seven patients had a history of previous IE. In all 7, the previous IE was secondary to a bacterial organism and was managed medically. No patients had a prior documented history of *Candida* infection at any site or a previous valve replacement. The median Pitt bacteremia score was 2 (Range: 0–5) and the median CCI score was 0 (Range: 0–6). Eleven patients had a history of IDU within 30 days of the index admission and 7 patients had positive hepatitis C RNA polymerase chain reaction testing on admission. Two patients had central venous catheters at the time their *Candida* infection was diagnosed, and 1 patient had a peripherally inserted central catheter. One patient required initiation of dialysis during their hospitalization. No other patients had a history of requiring dialysis.

All 12 patients met modified Duke and Duke-ISCVD criteria for definite IE. The median duration of bacteremia was 5 days (Range: 1–17 days). Eight patients had isolated bacteremia with *Staphylococcus aureus*. Five patients grew methicillin-susceptible *S aureus* and 3 patients grew methicillin-resistant *S aureus*. Three patients had polymicrobial bacteremia, and in all 3 instances *S aureus* was one of the pathogens. A single patient's cultures were positive for *Enterococcus faecalis*. Patients had a wide range of antibiotic exposures during their admission. Seven received broad-spectrum gram-positive therapy with vancomycin. Nine patients received broad-spectrum gram-negative therapy with cefepime, ceftaroline, ceftriaxone, ertapenem, or piperacillin-tazobactam. Two patients were treated with either nafcillin or cefazolin alone.

**Figure 1.**
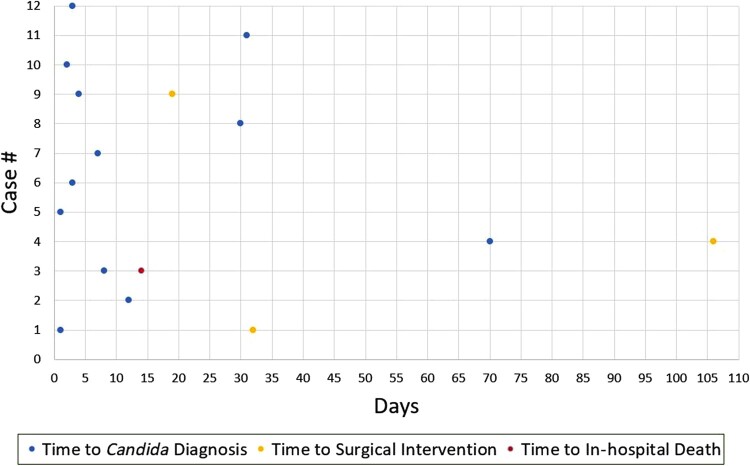
Scatter plot outlining the time to detection of the invasive *Candida* infection (blue), time to surgical intervention (yellow), and time to in-hospital death (red) for all 12 patients.

In 11 patients, candidemia was identified at a median of 4 days (Range:1–70 days; Figure 1). One patient developed *Candida* osteomyelitis at the site of a cranioplasty with bone cultures growing *Candida parapsilosis* but did not have a corresponding positive blood culture. The median duration of candidemia was 2 days (Range: 1–8 days). *Candida glabrata* was isolated in 4 patients and *C albicans* and *C parapsilosis* were isolated in 4 patients each, including 1 patient who grew both *C albicans* and *C parapsilosis.* One patient's blood cultures were positive for *Candida tropicalis*. The duration of antifungal therapy in patients who survived to discharge ranged from 14 days to indefinite therapy, which was recommended in 5 cases. One patient received only 6 days of antifungal treatment but passed away during the index hospitalization while on treatment. Eleven patients received micafungin at some point in their course. Three patients received liposomal amphotericin, 1 in conjunction with flucytosine. Nine patients were ultimately transitioned to oral therapy, 7 with fluconazole, 1 with voriconazole, and 1 with isavuconazole. Two patients underwent surgical valve replacement and 1 patient underwent open pulmonary embolectomy for a saddle pulmonary embolism from a large tricuspid valve vegetation. In 2 of these cases, surgical cultures (1 from a valve specimen and 1 from the pulmonary embolus) also grew the concordant *Candida* species.

Eleven patients survived to hospital discharge and 10 survived to 90 days postdischarge. The median time to last documented follow-up after the diagnosis of *Candida* infection for the 11 patients who survived to discharge was 532 days (Range: 65–830 days) and there were no documented relapses with *Candida*. Two additional patients died at 162 days and 555 days. One passed from a new episode of endocarditis with a bacterial organism and the other died from an opioid overdose.

## DISCUSSION

We report a case series of 12 patients with definite bacterial IE who developed invasive *Candida* infection manifested principally by candidemia. While *Candida* is a well-described cause of IE, most previous studies have described its role as the primary etiologic pathogen rather than an organism isolated during the course of treatment of bacterial IE. Although this is a retrospective case series, it highlights a concerning phenomenon with significant treatment implications that is affecting patients with bacterial IE. Notably, this outcome occurred in 5.1% of all patients with definite IE treated at our institution during the study period.

There were a number of similarities between the 12 patients in this series. Eleven reported IDU and 7 had prior bacterial IE. No patients had previously documented infections with *Candida*. In all but 1 case, *S aureus* was either the primary or, in the case of polymicrobial bacteremia, 1 of the primary pathogens. All 12 cases involved native valves, including 10 that affected the tricuspid valve. Additionally, 10 patients received broad-spectrum gram-positive or gram-negative antibiotic therapy in the days prior to detection of their *Candida* infection. Although there was a wide range with respect to the timing of candidemia, 8 patients had positive blood cultures for *Candida* within 7 days of starting antibiotic therapy.

Broad-spectrum antibiotic exposure and IDU are both known risk factors for invasive candidiasis [1, 8]. Several of the patients were also admitted to the intensive care unit and had indwelling central venous access, which increases the risk of candidemia [2]. However, 5 patients developed candidemia within 48 hours of admission. Recent literature has suggested that *S aureus* is able to invade host tissue and disseminate via adherence to hyphal elements of *C albicans*, suggesting that *Candida* could facilitate the invasion of *S aureus* in co-colonized patients [9]. It is possible that patients were colonized with both organisms and detection of *Candida* was delayed due to decreased sensitivity of blood cultures for detection of invasive candidiasis [10].

It remains unclear whether these episodes of candidemia led to superinfection of the valvular vegetations. Only 3 patients underwent surgical procedures with pathologic evaluation of valve or embolism specimens. Two of the 3 patients had positive operative cultures for *Candida* and these patients were candidemic for 6 and 8 days. In contrast, the patient with negative valve cultures and pathology only had 1 day of candidemia. Ultimately, 7 patients were believed to have *Candida* IE by the MDET, prompting recommendations for longer durations of treatment.

In our clinical practice, we do not routinely perform valve surgery during the index hospitalization for patients with tricuspid valve IE given the relatively low in-hospital mortality for this condition. Our approach advocates for treating the patients’ substance use disorder and having them return as an outpatient for evaluation for valve surgery when hemodynamic indications arise. Seven patients had isolated native tricuspid valve endocarditis and were managed accordingly. Of the 5 patients with left-sided endocarditis, 2 underwent valve surgery. In the other 3, 2 did not have surgical indications and 1 was deemed not a surgical candidate due to their neurologic condition (unresponsive wakefulness syndrome).

Another feature of this study was the relatively successful treatment with step-down oral therapy. Nine patients were transitioned to an oral triazole, including 7 who received fluconazole. These results are consistent with other previously published findings supporting the use of oral fluconazole in treatment of *Candida* IE [11]. Eleven patients survived to hospital discharge (8.3% mortality), which is substantially lower than the commonly reported 25% mortality associated with candidemia or 30% mortality seen with *Candida* IE [2, 12]. One additional patient passed away within 90 days postdischarge for an overall mortality of 16.7%.

## CONCLUSIONS

While additional data and prospective studies would add further validity to these observations, some tentative inferences can be drawn from this series. Patients undergoing treatment for bacterial IE, particularly individuals with IDU and *S aureus* bacteremia, may be at risk for candidemia, which can progress to superinfection of valvular vegetations. Clinical changes in patients, particularly those who inject drugs, undergoing bacterial IE treatment should prompt collection of blood cultures to evaluate for candidemia. A subset of patients may present asymptomatically, but the optimal surveillance strategy for detecting this complication remains unclear. Providers could consider repeating blood cultures 4–7 days after bacteremia clearance to assess for previously undetected candidemia. Finally, patients with candidemia, including *Candida* IE, can be successfully transitioned to oral azoles to complete therapy. Further research is needed to better understand the pathogenesis and optimal management of this phenomenon.

## Notes

***Patient consent.*** The design of this study has been approved by the University of Kentucky Institutional Review Board and per their approval, patient consent was not required.
